# The role of novel motor unit magnetic resonance imaging to investigate motor unit activity in ageing skeletal muscle

**DOI:** 10.1002/jcsm.12655

**Published:** 2020-12-22

**Authors:** Matthew G. Birkbeck, Andrew M. Blamire, Roger G. Whittaker, Avan Aihie Sayer, Richard M. Dodds

**Affiliations:** ^1^ Translational and Clinical Research Institute Newcastle University Newcastle upon Tyne UK; ^2^ NIHR Newcastle Biomedical Research Centre, Newcastle upon Tyne Hospitals NHS Foundation Trust, Faculty of Medical Sciences Newcastle University Newcastle upon Tyne UK; ^3^ Northern Medical Physics and Clinical Engineering Freeman Hospital, Newcastle upon Tyne NHS Foundation Trust Newcastle upon Tyne UK

**Keywords:** Sarcopenia, Skeletal muscle, Ageing, Motor unit, Electromyography, Magnetic resonance imaging

## Abstract

Sarcopenia is a progressive and generalized disease, more common in older adults, which manifests as a loss of muscle strength and mass. The pathophysiology of sarcopenia is still poorly understood with many mechanisms suggested. Age associated changes to the neuromuscular architecture, including motor units and their constituent muscle fibres, represent one such mechanism. Electromyography can be used to distinguish between different myopathies and produce counts of motor units. Evidence from electromyography studies suggests that with age, there is a loss of motor units, increases to the sizes of remaining units, and changes to their activity patterns. However, electromyography is invasive, can be uncomfortable, does not reveal the exact spatial position of motor units within muscle and is difficult to perform in deep muscles. We present a novel diffusion‐weighted magnetic resonance imaging technique called ‘motor unit magnetic resonance imaging (MUMRI)’. MUMRI aims to improve our understanding of the changes to the neuromuscular system associated with ageing, sarcopenia and other neuromuscular diseases. To date, we have demonstrated that MUMRI can be used to detect statistically significant differences in fasciculation rate of motor units between (*n* = 4) patients with amyotrophic lateral sclerosis (mean age ± SD: 53 ± 15) and a group of (*n* = 4) healthy controls (38 ± 7). Patients had significantly higher rates of fasciculation compared with healthy controls (mean = 99.1/min, range = 25.7–161.0 in patients vs. 7.7/min, range = 4.3–9.7 in controls; *P* < 0.05. MUMRI has detected differences in size, shape, and distribution of single human motor units between (*n* = 5) young healthy volunteers (29 ± 2.2) and (*n* = 5) healthy older volunteers (65.6 ± 14.8). The maximum size of motor unit territories in the older group was 12.4 ± 3.3 mm and 9.7 ± 2.7 mm in the young group; *P* < 0.05. MUMRI is an entirely non‐invasive tool, which can be used to detect physiological and pathological changes to motor units in neuromuscular diseases. MUMRI also has the potential to be used as an intermediate outcome measure in sarcopenia trials.

## Sarcopenia

Sarcopenia from the Greek ‘sarx’—flesh and ‘penia’—paucity, is a progressive and generalized degenerative muscle disease in which there is a loss of muscle strength and mass.^1^ Recently, the European Working Group on sarcopenia in older people published updated guidance indicating that low muscle strength and reduced muscle quantity and quality are now the primary criteria in the diagnosis of sarcopenia.^2^ It occurs most commonly in older age, but it is now recognized that it can occur earlier in the life course, for example, in association with long‐term conditions arising in mid‐life.^3^ Sarcopenia is important because it is associated with a number of adverse health outcomes such as falls, frailty, and increased mortality rates.^4^, ^5^, ^6^ It is estimated that in 50 years' time, more than 8.6 million people in the UK will be aged 65 and over,^7^ and hence, sarcopenia will become an ever more important issue. Understanding the underlying mechanisms will be key in developing robust diagnostic tools and targeted interventions.^8^

**Figure 1:**
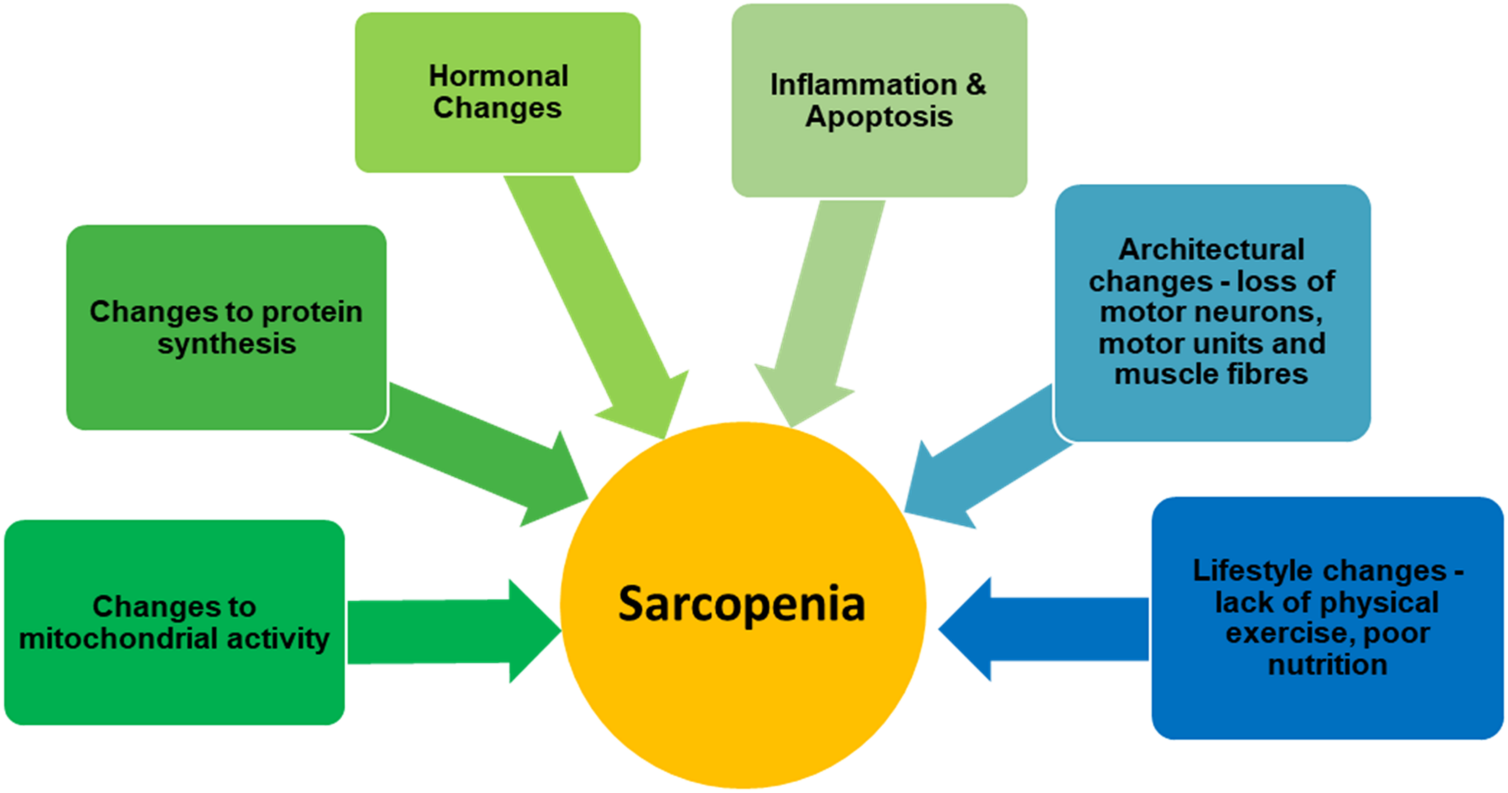
Currently established mechanisms underlying the development of sarcopenia.

Sarcopenia has a complex pathophysiology (*Figure* 1). Lifestyle risk factors for developing sarcopenia include a lack of physical exercise and poor nutrition.^9^ A range of biological mechanisms has been proposed for the development of sarcopenia including reduced mitochondrial function,^10^ changes to protein synthesis,^11^ hormonal changes, microvascular changes, satellite cell dysfunction, inflammation, apoptosis,^1^, ^12^ and changes to the neuromuscular system.^13^, ^14^ The aim of this review is to summarize evidence for changes to the neuromuscular system, in particular the human motor unit with age, which are considered major factors in the development of sarcopenia.^15^, ^16^ Furthermore, we discuss methods for assessing these changes including the use of novel magnetic resonance imaging (MRI) techniques.

### Architectural changes to the neuromuscular system

A motor unit consists of the anterior horn cell in the spinal cord, the myelinated axon and all muscle fibres, which that single axon innervates. Motor units can be categorized into two groups: Type I or ‘slow twitch’ motor units containing muscle fibres, which are rich in mitochondria and myoglobin. Fibres from these units contract at a slower speed, producing lower levels of force. Type II or ‘fast twitch’ motor units contain muscle fibres, which are not as rich in mitochondria or myoglobin and are able to contract with a higher speed, generating higher forces. Consequently, muscles which are composed of mainly Type I fibres are used to maintain posture whereas muscles which contain mainly Type II fibres are used for power movements such as pushing off when sprinting.^17^ There remains controversy over the hypothesis that as a person ages, Type II motor unit axons appear to be preferentially lost, with some human studies supporting this claim^18^, ^19^, ^20^ and another providing contrasting evidence.^21^ Furthermore, a specific effect on Type IIa fibres (fast twitch, oxidative) has recently been suggested.^22^

Although it remains a matter of conjecture whether a specific type of motor unit is preferentially depleted with age, it has been shown that surviving motor units can re‐innervate those which are lost.^23^, ^24^, ^25^ Re‐innervation processes increase the size of motor unit potentials (electrical activity from motor units), fibre density, and the grouping of fibre types.^26^ Eventually, denervation exceeds re‐innervation, and there is a progressive loss of motor units and muscle fibres.^16^, ^24^, ^27^, ^28^, ^29^ Primary endpoints of this process include a reduction in fine motor control, decline in skeletal muscle strength and mass, fatty infiltration of the remaining muscle, and a reduction in available twitch force generation (the force generated when a muscle contracts). Quantification of these changes to the motor units in ageing could therefore provide useful biomarkers to identify those at risk of developing sarcopenia, as well as intermediate outcomes for use in sarcopenia trials.

## Current techniques to investigate changes to the motor unit

Evidence that there is remodelling and eventual loss of motor units, changes in motor unit size (anatomically defined as the number of muscle fibres per motor unit^30^), changes to muscle fibre type, and reduction in twitch force are all potential biomarkers to stratify those patients at risk of developing sarcopenia. Currently, there are two main techniques to achieve measurements of such parameters: muscle biopsy and electromyography (EMG).

### Muscle biopsy

Structural changes to muscle tissue can be assessed through muscle biopsy and histological staining.^31^ Different choices of stain can be used to determine muscle fibre morphology (size of muscle fibres and regenerating and degenerating fibres), different fibre types, and obvious mitochondrial, glycogen, and lipid storage abnormalities.^32^, ^33^ Because of the small amount of tissue collected during the procedure, overt pathologies may be detected, whilst subtle changes associated with ageing or early disease may be overlooked. Biopsy is sensitive to the choice of site, for example, in an asymmetric disease, pathology may be missed if the biopsy site is chosen on the unaffected side. It is also an invasive technique with the potential for inter‐operator variability.

### Electromyography techniques

The current gold standard to investigate electrical activity from motor units and infer size, shape, and morphology is EMG. As a clinical technique, it has been in use since the 1960s.^34^ EMG is clinically useful because it can detect changes in muscle function before the development of weakness and wasting. This allows for the early diagnosis of a wide range of neuromuscular diseases including neuropathies, myopathies, and motor neuron diseases.^35^ Motor unit activity can be elicited either by voluntary contraction of the muscles or via electrical stimulation of the relevant nerve. This activity is recorded via surface or intramuscular electrodes. In humans, it is known that during voluntary activation, motor units are recruited in order of their size.^36^, ^37^ Small motor units have small diameter axons and innervate more fatigue resistant muscle fibres, whereas large motor units have large diameter axons and innervate fibres that are less resistant to fatigue.^38^ There are examples when recruitment order under voluntary activation has been shown to deviate from the size principle. In particular, when a muscle changes its action around a joint, for example, between abduction and flexion of the dorsal interossei, it has been shown that ~8% of motor unit pairs consistently reverse their recruitment order.^39^ This suggests that motor unit recruitment may be dependent on the type of movement performed rather than it being fixed for a particular muscle.

When considering electrical nerve stimulation, it is commonly stated that the recruitment order of motor units is reversed. This is primarily thought to be due to larger motor units displaying a lower threshold of electrical excitability and increased fatigue of motor units under electrical stimulation compared with volitional activity,^38^ as demonstrated in animal models.^40^ However, in humans, there is ongoing debate about this proposed reversal of recruitment order. Studies suggest that electrical stimulation produces randomized patterns of recruitment based on the method of stimulation (i.e. direct or indirect),^41^ geometry of the stimulating electrodes with respect to the nerve, and design of the stimulation experiment.^42^ The latter includes factors such as the current strength, the stimulation frequency, and the stimulation pulse duration. The strength of the stimulus applied to the nerve will affect the number of motor units recruited.^43^ Increasing the frequency of stimulation will increase the force production; however, this leads to increased muscle fibre fatigue.^44^ The length of the delivered stimulus pulse has been correlated with motor unit activity, and it has been shown that as the pulse length increases, a lower stimulus intensity is required to produce activity.^38^ In summary, the design of experiments implementing electrical stimulation to study motor unit recruitment order and activity needs careful consideration.

#### Surface electromyography

Surface EMG is cheap and is readily available. Because the recording electrodes are relatively large (typically several millimetres diameter) a large number of motor units can be recorded. This is advantageous for estimating the number of motor units in a muscle and their recruitment pattern. One example of a method using only surface EMG to measure motor unit activity is high‐density surface EMG. This is a non‐invasive method offering the possibility to measure single‐motor unit potentials and provide an estimation of the numbers of motor units in muscles.^45^ However, due to attenuation of the stimulus by subcutaneous tissues, electrical activity is detected only from motor units within 10–12 mm of the muscle surface (10–12 mm depth),^46^ leading to an underestimation in the number of active motor units. Furthermore, because the recorded signal is a spatially and temporally averaged composite of all the muscle fibre action potentials within the pick‐up area of the electrode, changes in muscle fibre distribution that occur in sarcopenia are largely undetectable.

#### Needle electromyography

Needle electrodes allow intramuscular assessment of motor unit activity and can therefore access motor units that are located deeper within the muscle. Recordings are made from a small area at the tip of the needle with a hemisphere of radius ~1 mm: within this volume, there are hundreds of muscle fibres. Muscle fibres from motor units are interdigitated within muscles; therefore, the sensitive region of the electrode may contain between four and six fibres from a single‐motor unit.^35^ Needle EMG can be used to detect useful diagnostic information such as firing rates and potentials of single or multiple motor units. However, the needle has to be inserted at multiple sites in the muscle, and this can be uncomfortable for some patients. There are also a number of variables that can affect the amplitudes and shapes of the EMG potentials and may introduce artefact and error into the measurements including electrode placement, subject co‐operation, and body habitus.^47^

#### Morphology of motor units

Needle EMG provides useful information on the muscle fibres adjacent to the electrode, but because a single recording surface is used it provides no information as to their distribution in space. Motor unit morphology (i.e. size and shape of muscle fibre territories) can be studied using techniques such as scanning EMG, which involves initial insertion of a needle electrode into the muscle to locate motor unit activity after which the needle is drawn backwards through the territory of electrical activity to map the distribution of the unit. This technique is very time consuming, samples only a single‐motor unit at a time, and has never entered routine clinical practice.^48^, ^49^ These techniques have however been applied to study the morphology of motor units in a small number of human studies. Data show that motor unit territories are between 5 and 15 mm in diameter.^50^, ^51^

Scanning EMG also provides supporting evidence to glycogen depletion studies performed in animals.^52^, ^53^ Glycogen depletion requires isolation and prolonged stimulation of a single motor axon such that the glycogen stores in the muscle fibres that this axon innervates are selectively depleted. The muscle is then sectioned and stained with a Periodic acid–Schiff technique to highlight the glycogen depleted fibres. These studies show that motor units are interdigitated within muscle, shown by clusters of muscle fibres across the muscle innervated from a single‐motor unit. Scanning EMG has detected the presence of ‘silent zones’ between clusters of muscle fibres, further supporting the hypothesis that motor units are not homogenously distributed in muscle because of the branching nature of motor axons.^54^

#### Motor unit number estimation

The technique of motor unit number estimation (MUNE) was developed by McComas *et al*.^55^ to increase the quantitative data available from EMG. There are multiple MUNE techniques, which have been developed as an extension of McComas' work, and we describe incremental stimulation. Other techniques include multiple point stimulation, high‐density surface EMG, spike‐triggered averaging, motor unit number index, and intramuscular MUNE. Basic principles, advantages and disadvantages, and references for further reading for each of these techniques are provided in *Table* 1.

**Table 1:** Overview of electromyography techniques to perform motor unit number estimation (MUNE)

MUNE Technique	Principle	Advantages	Disadvantages	References
Incremental current stimulation (ICS)	Use surface or needle electrode to stimulate nerve of interest at a single site. Stimulation is performed at multiple incremental currents. Record CMAP and SMUPs.	• Relatively simple to perform.	• Motor unit alternation will lead to overestimate in number of motor units.	^55^
Multiple point stimulation (MPS)	Use of surface or needle electrode to stimulate nerve of interest at multiple sites. Stimulation is performed at multiple incremental currents. Record CMAP and SMUPs.	• Reduces influence of alternation due to the use of more than one stimulation site.	• Time consuming due to need to stimulate multiple sites.	^56^
High‐density surface EMG	Use of surface electrode grid to cover large area. Record CMAP and SMUPs from multiple sites, simultaneously.	• Individual motor unit potentials can be extracted more easily due to multiple channels. Allows for simultaneous measurement of activity in multiple locations. • Prevents same motor unit being counted more than once in SMUP. • Uses voluntary activation of muscles. • Allows measurement of MFCV	• Use of surface EMG—signal is composite of potentials. • Insensitive to deep muscles.	^45^, ^57^
Spike‐triggered averaging (STA)	Use of needle electrode for intramuscular stimulation. Recordings are triggered from SMUPs. Record CMAP. Record multiple SMUPs and average.	• Can be applied to both proximal and distal muscles. • Effect of alternation is removed.	• Requires needle insertion. • Considerable patient co‐operation required as lengthy examination.	^58^, ^59^
MUNIX	Use of surface or needle EMG to record CMAP. Record interference pattern produced by voluntary activation to derive SMUPs. A model is then fitted to the data to derive the MUNE.	• Reduced number of electrical stimuli as SMUPs recorded using voluntary activation.	• Requires software for data analysis, code for which is not open source, however equations are provided.	^60^, ^61^, ^62^, ^63^
iMUNE	Use of intramuscular needle to record SMUPs, averaged into mean SMUP. Measurement of CSA of the muscle. iMUNE value is mean MUP divided by CSA.	• Accounts for size of muscle. • Can sample at different depths to account for changes in unit size with depth.	• Requires needle insertion. • Requires measurement of muscle CSA.	^29^

CMAP, compound muscle action potential; CSA, cross‐sectional area; EMG, electromyography; iMUNE, intramuscular motor unit number estimation; MFCV, muscle fibre conduction velocity; MUNE, motor unit number estimation; MUNIX, motor unit number index; SMUP, single‐motor unit potential.

An in depth review of EMG techniques can be found at de Carvalho *et al*.^64^

The incremental stimulation method applies an electrical stimulus is delivered to the motor nerve, sufficient to activate all the motor units within the muscle of interest. The amplitude of the resulting action potential is recorded, typically using surface EMG electrodes. This is the compound muscle action potential and represents a superposition of all fibre depolarizations within the recording range of the selected electrode.^65^ Next, incremental current nerve stimulation is used to record multiple single‐motor unit potentials (SMUPs), which are observed as the stimulus current reaches the threshold for each motor unit. The number of motor units is then given by the following equation^55^, ^66^, ^67^:

(1) $$\text{MUNE}=\frac{\text{CMAP amplitude}}{\text{average SMUP amplitude}}$$

One of the major limitations of this method is a phenomenon known as alternation. Alternation occurs when two or more motor unit axons have overlapping activation thresholds (the current at which the motor unit responds to stimulus and discharges). Therefore, more than one different combination of motor units may respond for a given stimulus level.^45^ It is thought that alternation leads to an overestimation in the number of motor units as the same motor unit may contribute to the compound muscle action potential more than once.^68^ A second limitation is that only motor units within 5–10 mm of the electrodes are detected, making it impossible to study deep muscles.

There are some practical issues concerning MUNE, which apply to all of the techniques mentioned earlier. Careful setup of equipment and performance of the study is required to produce reliable and reproducible results.^64^ Subject tolerance must also be considered; for example, motor unit number index^60^ may be preferred over spike‐triggered averaging^58^ or multiple point stimulation^56^ due to patient compliance as it is a quicker technique. In summary, MUNE is a useful tool to estimate motor unit numbers in muscles and can be used as a surrogate biomarker in neuromuscular disorders. It has demonstrated a reduction in the number of motor units in a number of ageing studies as described later. However, careful interpretation of the results is required, and there are some disadvantages to each of the methods proposed.

### Electromyography in studies of ageing and sarcopenia

Electromyography has been used in innovative ways in a large number of studies to investigate the effect of ageing on the neuromuscular system. Consistent findings include a relationship between increased age and a decrease in the number of active motor units, concurrent with an increase in the number of fibres a given motor unit innervates.^69^ Piasecki *et al*.^24^, ^70^ investigated surface motor unit potentials (sMUPs) and intramuscular motor unit potentials (imMUPs) in four groups of male patients: healthy young (mean age: 26.6 years), non‐sarcopenic older (mean age: 68.4 years), pre‐sarcopenic older (mean age: 72.6 years), and sarcopenic older (mean age: 74.3 years). They found that the amplitude of sMUPs in the vastus lateralis muscles did not differ between groups, whilst imMUPs were significantly smaller in the sarcopenic group compared with those in the pre‐sarcopenic group. In the tibialis anterior, sMUPs and imMUPs were larger in all older groups compared with younger groups; however, they were not significantly different between older groups. These data suggest that there is a heterogeneity in the development of sarcopenia between individual muscles and that the use of intramuscular EMG to detect changes between these groups is more sensitive than surface EMG. Differences between older non‐sarcopenic and sarcopenic groups suggest that a failure to recompensate for the loss of muscle fibres in sarcopenic individuals, which is normally performed by re‐innervation from surviving motor units, may be a driver in the development of the disease. Both of these findings provide useful information about the aetiology of sarcopenia.

The ability of a muscle to produce force is dependent on its fibre type composition and the contractile velocities of those fibres. High‐density surface EMG can be used to measure the propagation velocity of the motor unit action potential along the sarcolemma. This is called the muscle fibre conduction velocity (MFCV), which is positively related to the size of the recruited fibres.^71^ This method involves using a grid of multiple electrodes to record activity entirely non‐invasively. Measurements of MFCV can also be performed by inserting two needle electrodes at a set distance apart along the muscle fibre axis. One of the electrodes acts as a stimulating electrode innervating a small bundle of muscle fibres. Action potentials from these fibres are detected using the recording needle electrode. To ensure the action potentials are from a single‐motor unit, at least five identical potentials are needed. The time to peak voltage of these action potentials is measured. MFCV is then derived by dividing this time into the distance between the electrodes. Boccia *et al*. demonstrated velocities measured in the vastus medialis were significantly less affected by ageing than those in the vastus lateralis.^72^ These data are further supported by histochemical studies showing a significantly higher population of type II muscle fibres in the vastus lateralis compared to the vastus medialis.^73^ This again suggests that different muscles may undergo changes to motor unit composition at different rates and time‐points across the life course.^74^, ^75^, ^76^

Decreases in the number of MUs show a stronger correlation with age than the size of the motor unit potential.^24^, ^27^, ^72^, ^77^, ^78^, ^79^, ^80^, ^81^ These studies concluded that numbers of motor units are significantly higher in younger groups than older; however, there is evidence that between groups of older male patients (e.g. non‐sarcopenic older vs. sarcopenic older) differences in motor unit number are difficult to detect using current MUNE techniques.^70^ This suggests that although MUNE can detect differences between young non‐sarcopenic individuals and older sarcopenic individuals, it is not sensitive enough to distinguish those who may be at risk of or who are already developing sarcopenia.

There are a number of areas where the current techniques discussed fall short in the investigation of motor unit activity in older people: limited sensitivity of EMG measures to subtle changes in motor unit activity associated with ageing across the life course, difficulty assessing motor unit activity in multiple and deep muscles, and assessing older people living with frailty using currently available invasive procedures. Therefore, there is a need to develop a sensitive and quantitative technology, to determine the relationship between motor unit changes and the development of sarcopenia in individuals. This will improve our understanding of the heterogeneity of sarcopenia and allow better monitoring of responses to tailored interventions.

In recently published work,^82^, ^83^ our group has described, developed, and applied a novel MRI technique on the basis of diffusion‐weighted imaging (DWI), which allows us to non‐invasively assess motor unit activity in an entirely new way. We call this technique ‘motor unit MRI—MUMRI’. The following sections of this review will cover the basic principles of DWI, a discussion of the findings to date using MUMRI and potential future applications of this technique.

## Diffusion Weighted Magnetic Resonance Imaging

### Basic concepts in diffusion MRI

Magnetic resonance imaging (MRI) is based on the properties of the hydrogen nuclei (^1^H) within all water molecules, which possess a small magnetic moment. In the external applied magnetic field of the MRI scanner, the magnetic moments of all water molecules align and produce a weak net magnetization. During an MRI measurement, we manipulate this magnetization through the application of radiofrequency pulses to the area of the body we are imaging and by introducing spatiotemporal variations in the magnetic field across the same area of the body. These variations are called magnetic field gradients, and their application allows us to vary the frequency and phase of the MR signal arising from each ^1^H nuclei through which we encode the spatial location of the water molecules within the imaging volume. At a given time during the experiment, we sample these data in a specific slice of the volume; we then apply a mathematical Fourier transformation to these data converting it into an MR image. In a single imaging voxel (a 3D volume element of an image), the intensity of the MR signal produced is first proportional to the amount of hydrogen nuclei in that voxel, but then can also be linked to (or ‘weighted’ by) other biologically important features of the tissue microenvironment. One such image weighting links image intensity to the diffusion characteristics of the tissue.

**Figure 2:**
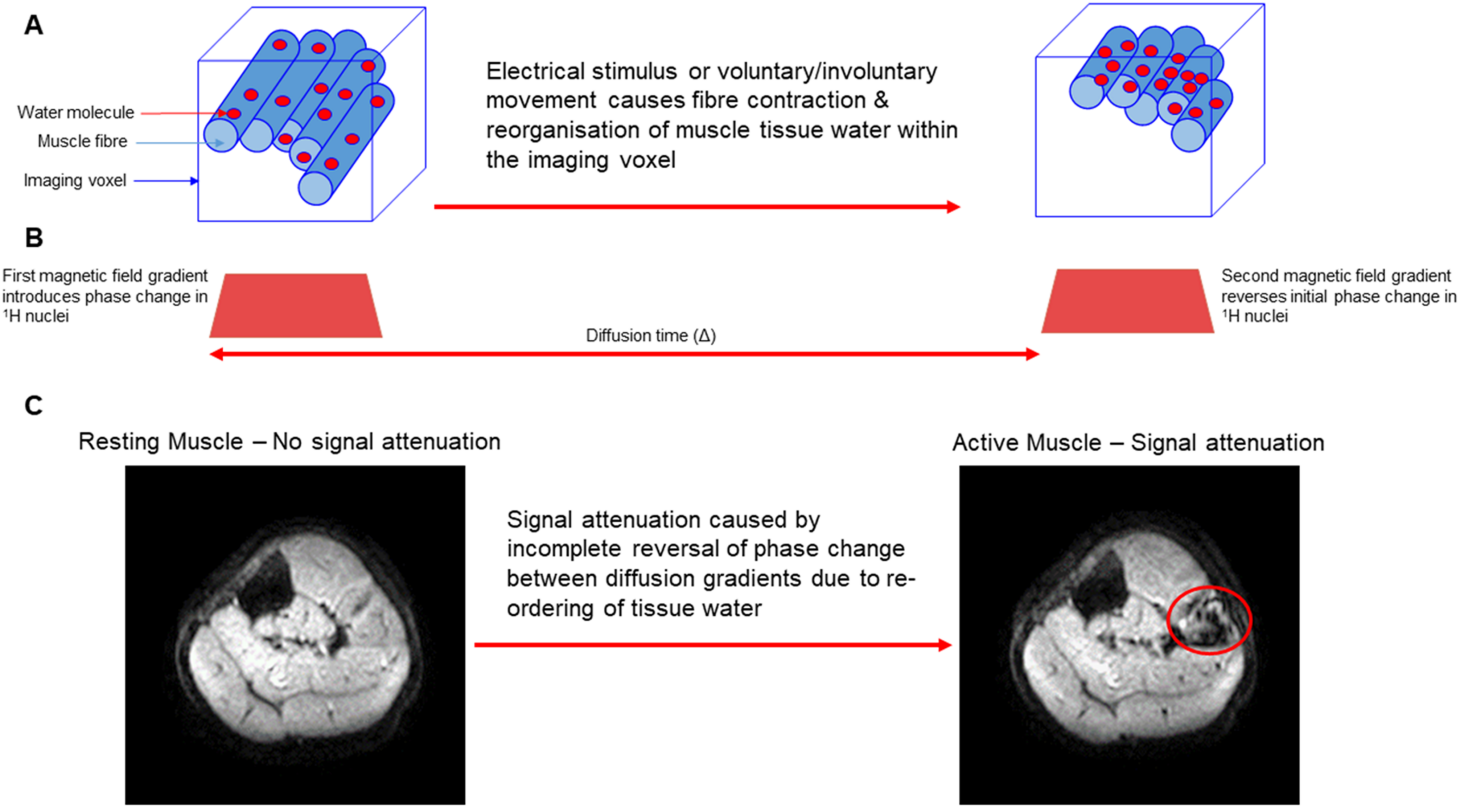
Schematic depicting the proposed mechanism of motor unit magnetic resonance imaging (MUMRI) image contrast. (*A*) Muscle fibres and constituent tissue water molecules are shown within the imaging voxel. Because of an electrical stimulus or voluntary/involuntary activation of a motor unit, muscle fibres will contract, re‐organizing the tissue water molecules. (*B*) During a diffusion‐weighted magnetic resonance imaging experiment, the first gradient introduces a phase shift in the ^1^H nuclei within the water molecules. The second gradient reverses this effect. However, because of the re‐organization of water molecules, this phase shift is not completely reversed. (*C*) This incomplete reversal of the phase shift leads to a signal attenuation visible on MUMRI images. The area of motor unit activity is indicated by red circle on right hand MUMRI image. It is important to note that this signal attenuation is also present when the muscle fibres relax, as relaxation of muscle fibres also causes a re‐organization of tissue water molecules.

Diffusion is the random motion of molecules in a fluid driven by their thermal energy, known as Brownian motion. In free water, the displacements of molecules are isotropic (i.e. have the same probability in all directions). In biological tissues, there are many barriers to free diffusion (e.g. cell membranes). These barriers restrict the diffusion of water molecules, and as such, the distribution of displacements becomes ‘non‐Gaussian’ and directionally non‐uniform (anisotropic), reflecting the organizational microstructure of the tissue through which the water is passing. To sensitize the experiment to the diffusion of water molecules and reveal this microstructure, we apply an additional pair of magnetic field gradients to the experiment (*Figure* 2). The first gradient introduces a phase shift in the MRI signal dependent on the position of the hydrogen (^1^H) nuclei in the magnetic field. The scanner then waits a defined time (Δ), allowing diffusion of ^1^H nuclei before applying a second identical gradient to reverse this phase shift. If there is diffusion of water molecules occurring during the interval between the two gradients, the second gradient will not completely reverse the phase shift introduced by the first, and there will be an overall net phase change. This phase shift results in an attenuation of the MR signal. Therefore, contrast in diffusion‐weighted images (DWI) is affected by the rate at which diffusion occurs in different tissues. For relatively ‘free’ diffusion there is a large net phase change resulting in high signal attenuation. These areas appear dark in a diffusion‐weighted images. In areas of ‘restricted’ diffusion, the net phase change is lower resulting in a smaller signal attenuation. These areas appear bright in diffusion‐weighted images.

### Diffusion magnetic resonance imaging to detect motor unit activity

Although these diffusion sequences are designed to detect diffusion of water molecules, any process that rearranges the *relative* spatial position of these molecules within the imaging voxel will produce signal attenuation, including when muscle fibres contract or relax (*Figure* 2). In 2015, Steidle and Schick^84^ described spontaneous signal voids of 5–15 mm diameter seen in DWI of healthy resting calf muscle. They termed these voids spontaneous mechanical activities of the musculature. However, shortly after Szeverenyi and Bydder^85^ introduced the concept of these as fasciculations, that is, spontaneous motor unit contractions.

Recently, our group postulated that the contrast mechanism in these scans is due to the effective intra‐voxel reorganization of tissue water caused by the contraction and relaxation of muscle fibres. This presents a novel contrast mechanism and a method for detecting the muscle fibre contraction associated with firing of the motor unit (*Figure* 2). We refer to the use of diffusion weighting in this way as motor unit MRI (MUMRI), and the method can be used to study both spontaneous and stimulated activation of motor units.

## Applications of motor unit magnetic resonance imaging

### Neuromuscular pathologies

In neuromuscular disease, the MUMRI technique has been applied in a small group (*n* = 4) patients with amyotrophic lateral sclerosis (ALS) (mean age ± SD: 53 ± 15 years), comparing these (*n* = 4) healthy controls (mean age ± SD: 38 ± 7).^83^ For this study, subjects were imaged at rest to observe fasciculation, which is a clinical hallmark of ALS. Patients had significantly higher rates of fasciculation compared with healthy controls (mean = 99.1/min, range = 25.7–161.0 in patients vs. 7.7/min, range = 4.3–9.7 in controls; *P* < 0.05; supporting information, *Videos* S1 and S2). We believe that this is the first reported technique to provide information on spatial and temporal patterns of motor unit activity in multiple muscles simultaneously. It also suggests that MUMRI may provide useful biomarkers in the detection of neuromuscular disease.

Fasciculation of an individual motor unit is relatively infrequent, and when using needle EMG, it is necessary to record for at least 30 s to be certain of detecting an event.^35^ In contrast, dozens of events were detected using MUMRI in the equivalent period. A second advantage was that unlike surface EMG; fasciculation was equally likely to be detected in deep as in superficial muscles. This suggests that MUMRI may be more sensitive than conventional electrophysiological techniques. However, further comparative studies with larger patient populations are needed to demonstrate if MUMRI could be an earlier prognostic indicator in the diagnosis of motor neuron diseases.

At present, we have applied MUMRI to the lower leg muscles as the direction of the muscle fibres is well known.^86^ This aided the choice of our optimal diffusion gradient sensitization. In principle, MUMRI can be extended to any region of the body including those with complex fibre arrangements such as paraspinal muscles and the tongue, the latter being crucial in the development of ALS. However, these muscles come with complexities both from an imaging and stimulation standpoint. We are currently in the early stages of applying MUMRI to other muscle groups, beginning in the upper limbs we are aiming to develop imaging protocols for specific muscle groups by optimizing the choice of imaging coil, MRI parameters, acquisition time, participant comfort, and stimulation protocols. This will allow us to work towards a whole‐body MUMRI protocol.

### Size, shape, and distribution of human motor units

**Figure 3:**
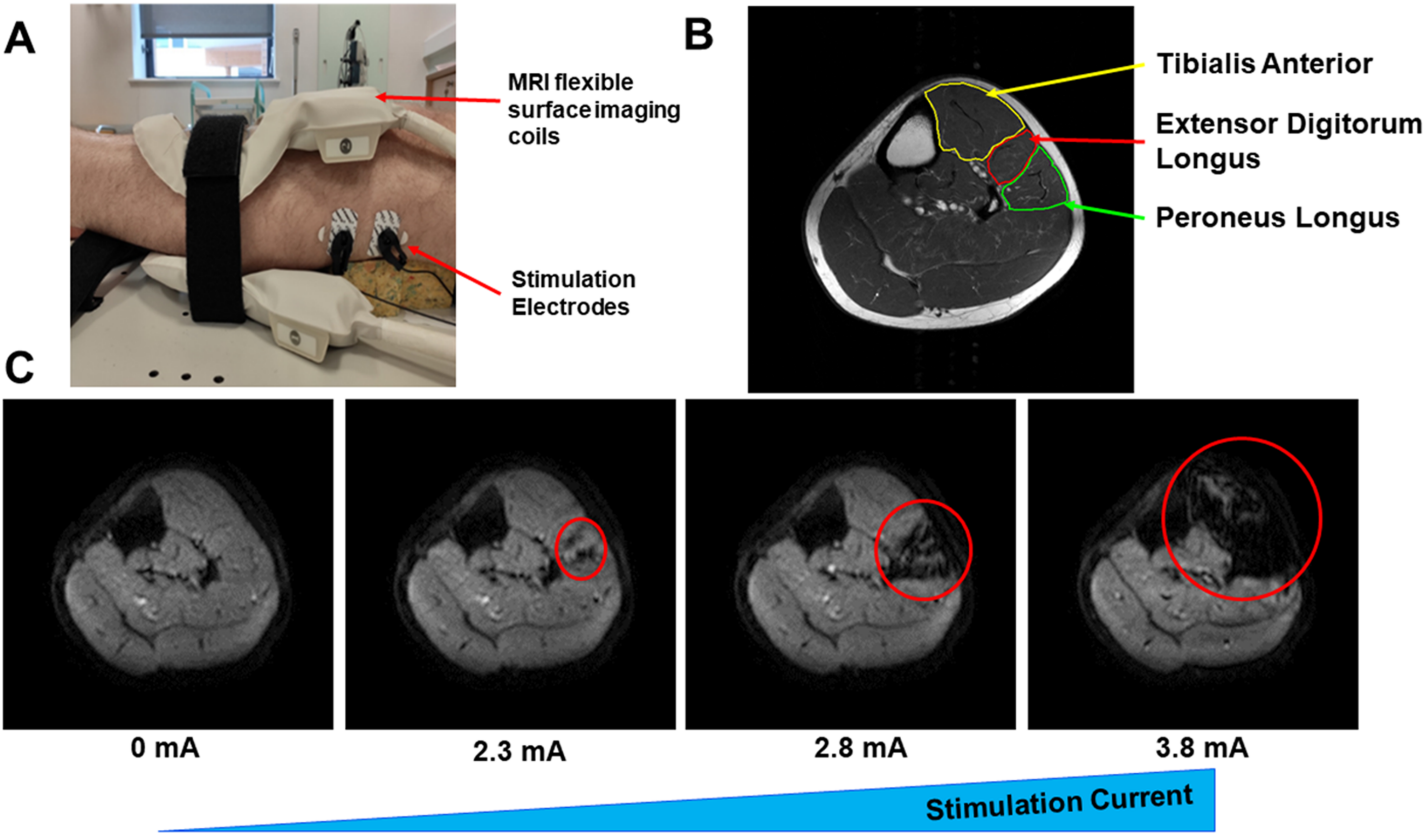
An example of the motor unit magnetic resonance imaging setup and resulting images. (*A*) Example experimental setup for stimulation of the fibular nerve which innervates muscles of the anterior compartment. (*B*) Axial T1w magnetic resonance imaging of the lower leg muscles, muscles of the anterior compartment are indicated (yellow—tibialis anterior, red—extensor digitorum longus, and green—peroneus longus). (*C*) Four axial diffusion‐weighted images of the lower leg muscles. Left image is with no stimulus applied, and the volunteer is at rest. As the stimulus current is increased, motor unit activity indicated by the red circles can be observed beginning in the *extensor digitorum longus* at 2.3 mA; eventually, the whole anterior compartment has been activated at 3.8 mA.

**Figure 4:**
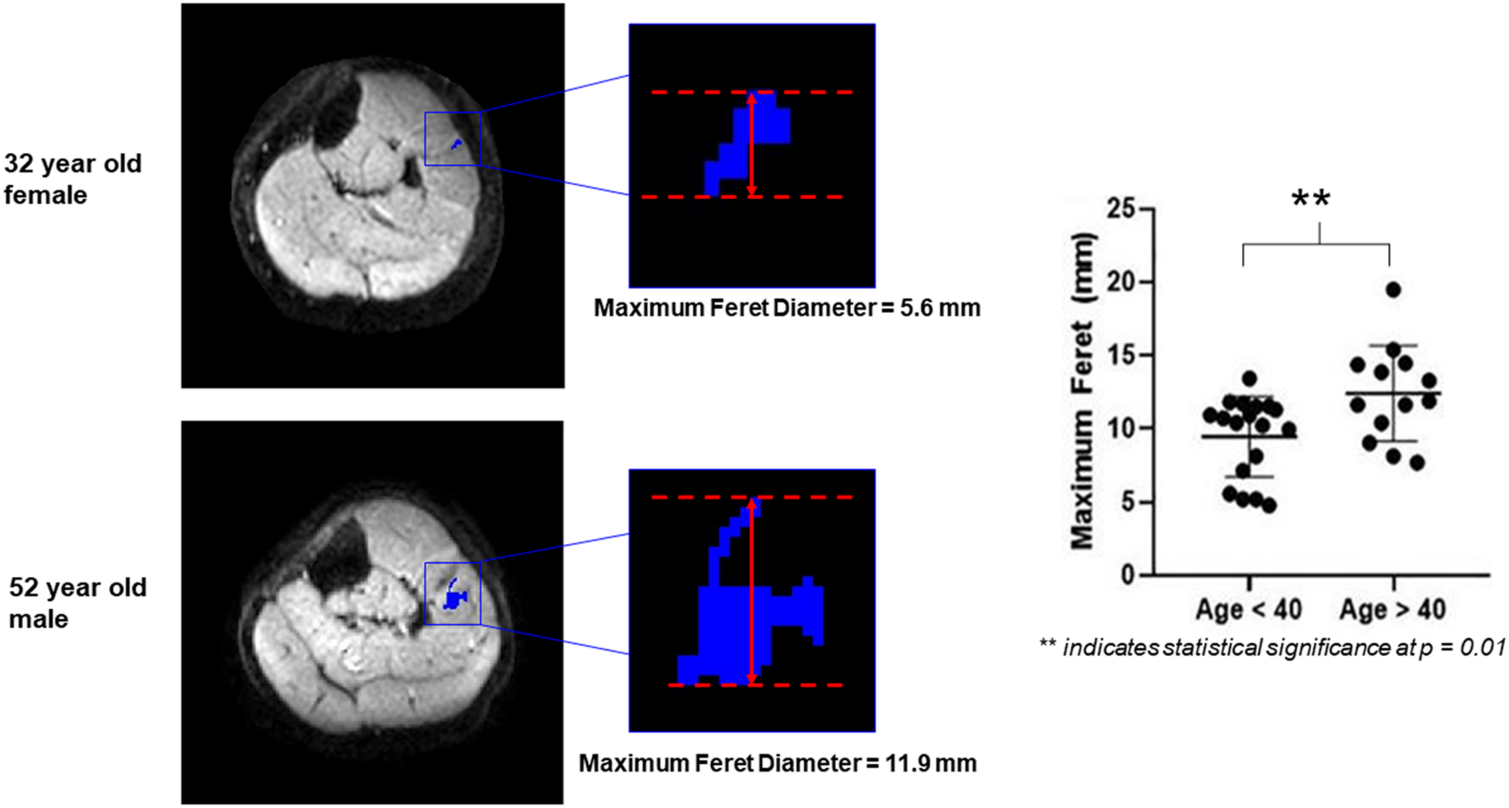
Left—example colour overlays of two areas of motor unit activity in two volunteers undergoing motor unit magnetic resonance imaging (top: 32‐year‐old female, bottom: 52‐year‐old male) from Birkbeck *et al*.^82^ Zoomed images centrally depict how to measure the maximum Feret diameter. Right—scatter plots showing statistically significant difference between maximum Feret dimensions of motor units for volunteers aged <40 compared with those >40. Reproduced with permission from Birkbeck *et al*.^82^

Our group has also applied MUMRI to study, in detail, the shape, size, and distribution of single human motor units in the lower leg muscles.^82^ Using in scanner electrical stimulation (*Figure* 3), it is possible to determine the firing thresholds of single‐motor units, observe alternation characteristics, and measure the size, shape, and distribution of motor units in multiple muscles simultaneously. Previously measuring these characteristics has only been possible using invasive techniques and hence only available in animal models. We found that between two groups of healthy volunteers split by age at 40 (Group 1: 5 volunteers, mean age ± SD: 29 ± 2.2 years. Group 2: 5 volunteers, mean age ± SD: 65.6 ± 14.8 years) that the mean maximum Feret dimension of the areas of motor unit activity (defined as the distance between two parallel planes restricting the object) was significantly higher in the older group (12.4 ± 3.3 mm) than the younger group (9.7 ± 2.7 mm); *P* < 0.05. An example of these data is shown in *Figure* 4. A further interesting finding was the observation of two ‘split’ motor units: areas of activity which alternated at the same time but were spatially distinct from each other and were therefore considered to be from the same motor unit. Silent zones (areas of motor unit inactivity where the peak amplitude drops below 50 μV p–p) have been observed in scanning EMG transects.^51^ It is possible that these areas correspond to the ‘split’ motor units we have observed, suggesting that motor units in humans are more complex than lower organisms. From our data, we are unable to show if these areas re‐group to form single structures at different points along the muscle. To investigate this, we will employ MUMRI in a multi‐slice protocol and acquire sagittal or coronal images.

This work employed a highly conservative approach to identifying if a motor unit as truly ‘single’. We chose only spatially discrete regions of activity for our analysis. As such this limited the yield of motor units as areas containing overlapping units were excluded. We are currently developing analysis pathways to increase our motor unit yield and are considering the use of voxel‐wise cross‐correlation between active regions and the implementation of Gaussian mixed models to identify concomitant regions of activity.

Motor units interdigitate with many others in muscles; therefore, a question arises as to why one single‐motor unit would produce such a discrete and well‐subscribed signal loss in our MUMRI images. Due to the close mechanical coupling of adjacent motor units, it could be that the observed signal voids overestimate the true area of a single‐motor unit.^82^ On the contrary, there may be too few muscle fibres in voxels at the periphery of the active region to produce an observable signal change. Currently, we do not know the combined effect of these two errors on our estimates of size of single‐motor units. In an attempt to answer this question, we are employing a phase‐contrast (PC) MRI (PC MRI) sequence, conventionally used to look at blood flow. This allows us to interrogate the motor unit territory and provides an estimate of muscle twitch velocity in centimetre per second. This will allow us to demonstrate if there are inactive fibres that are translated or ‘pulled along’ by the active muscle fibres and compare this region with a region from a diffusion‐weighted image. To provide a definitive answer, MUMRI would need to be performed *in vitro* alongside glycogen depletion to map the exact size of a single‐motor unit.

Although this study presented some limitations and contained a small sample size, these data support the hypothesis that older individuals have larger motor units and that age‐related motor unit re‐modelling is non‐uniform. Furthermore, these initial findings suggest that MUMRI is able to detect physiologically relevant changes to the motor unit associated with age. It is important to note that if in the future, MUMRI were to be used as a diagnostic technique for sarcopenia it would require validation against current standardized tools available such as: grip strength, chair rise time for assessment of muscle strength and gait speed to assess physical performance.^2^ Currently, we have a study underway, which is applying MUMRI to a larger group of approximately 60 individuals aged 45–85 years. In this study, we are also implementing clinically relevant tests for sarcopenia such as grip strength and chair rise time alongside MUMRI. As one part of this study, we aim to repeat the techniques used in Birkbeck *et al*.^82^ to quantify changes to motor unit activity in a larger ageing population.

### Biomechanical properties of muscle and motor unit type

Muscle fibre contraction time is a useful biomarker in studies of ageing populations. PC MRI can be used to study muscle fibre contraction velocity characteristics. By applying the stimulus at different times relative to our sequence, we are able to map out the muscle twitch^83^; this is known to differ between Types I and II motor units. We hope to link this with the velocity data from PC imaging, allowing for an estimate of the type of motor unit being studied.

Diffusion‐weighted MRI sequences can be designed to be sensitive to diffusion in a single direction or many. It is not yet known how the choice of diffusion sensitivity direction effects the contrast observed in MUMRI images. We postulate that when the direction of the fibre and diffusion sensitivity are the same, then the observed contrast will be greatest. We therefore propose that MUMRI can be extended to a diffusion tensor imaging approach. Diffusion tensor imaging exploits the anisotropy of diffusion in tissue and can provide information about the orientation of muscle fibres. A systematic study of diffusion gradient orientation and fibre direction for different muscles would be required to fully answer these questions.

We have developed an in‐scanner force transducer, allowing investigation of motor unit activity under voluntary contraction of muscles. This advances the technique as it is not only more physiologically accurate due to the differences in recruitment order of motor units between electrical and voluntary activation but also removes the need for potentially uncomfortable electrical stimulation, making it more clinically feasible and acceptable to patients. However, during voluntary muscle contraction, motor units fire at unpredictable times within the imaging window, making it more challenging to detect and map out the activity of individual motor units.

### Combined technologies and interventions

Motor unit magnetic resonance imaging can be used as a tool to guide EMG procedures. We are currently developing a pathway allowing MUMRI images to be fused with real time ultrasound to guide needle EMG, with the endpoints of tracking changes to the same population of motor units over time and to improve the accuracy of EMG targeting. Finally, there is scope to use MUMRI as a monitoring tool to observe the effects of exercise, diet, and drug interventions (e.g. resistance exercise, dietary interventions, and drugs) in sarcopenia trials.

### Challenges of the motor unit magnetic resonance imaging technique

There will undoubtedly be challenges associated with applying MUMRI in patients with sarcopenia and neuromuscular disorders. Patient‐specific challenges may include the ability of patients living with frailty to undergo MR examinations, which may include electrical stimulation, patients who have contraindications to MR (e.g. pacemakers or spinal cord stimulators), and adaptation of current lower limb MR protocols to other areas of the body, which may be difficult to image, for example, the arm muscles which are commonly affected in sarcopenia. Technical challenges are likely to include automating the analysis of data to provide a rapid and robust software package for clinical use; this will require input from a multidisciplinary team including MRI physicists and neurophysiologists. Clinical challenges include relating MR measures to current clinical measures (e.g. MUNE) and how to implement MUMRI measures as quantitative outcome measures in clinical trials.

It is important to note that as a technique, MUMRI can provide additional information to the field of neurophysiology such as the exact spatial locations of motor unit activity and size and shapes of single‐motor unit territories. However, we believe that MUMRI is not intended to replace EMG and should be used as a parallel tool in conjunction with standard techniques to add to the rich data that these techniques already provide, integrating past and future ideas to improve our understanding of motor unit pathophysiology.^87^

## Conclusions

Motor unit magnetic resonance imaging is a unique non‐invasive technique that provides a previously unattainable view of motor unit structure. There remain unanswered and interesting questions, which need to be answered to unlock the full potential of the MUMRI technique. We hope that studies we have underway will attempt to do so. We believe that this approach could be used to provide potential imaging biomarkers to distinguish between healthy, ageing, and pathological muscle as well as monitor disease progression over time in conditions such as sarcopenia, ALS, and spinal muscular atrophy. As such, MUMRI has the potential to revolutionize studies in skeletal muscle ageing, sarcopenia, and neuromuscular disorders.

## Funding

This work is being supported by the Medical Research Council Confidence in Concept (CiC) award (Newcastle University study number 1621/7484/2018), Muscular Dystrophy UK (grant number: 18GRO‐PG36‐0246‐1), and NIHR Newcastle Biomedical Research Centre (Doctoral Fellowship award to MB).

## Conflicts of Interest

Matthew Birkbeck, Andrew Blamire, Roger Whittaker, Avan Sayer, and Richard Dodds declare that they have no conflicts of interest.

## Supporting information


**Video S1**

**Video S2**
